# Reviews

**Published:** 1922-04

**Authors:** 


### REVIEWS OF BOOKS.

**THE CAUSATION OF SEX IN MAN.** By E. Rumley Dawson, L.R.C.P., M.R.C.S. Third edition. Illustrated. (H. K. Lewis & Co.; 7s. 6d. net.)

There are many notable instances in our profession of the sympathetic interest of the wife in her husband's life-work. In the third edition of "The Causation of Sex in Man" the preface written by Mrs. Rumley Dawson and the evidences of revision of the text are testimony of her perfect acquaintance with the subject, and, as she herself says, of the confidence she shared with her late husband that science will one day establish the truth of the thesis he so vigorously maintained. For over thirty years Dr. Rumley Dawson had given untiring energy to the study of the subject, and in 1900 what at the time was called "a very remarkable monograph" was read by him before the Obstetrical Society of London. The paper was entitled "The Essential Factor in the Causation of Sex: a New Theory of Sex"; and the occasion, strange to say, was the first on which the subject had ever been discussed by the Society. At that time

*An Essay on the History of Electrotherapy and Diagnosis. By Hector A. Colwell, M.B. Pp. 180. Illustrated. (Heinemann, 17s. 6d. net.)

the author supported his contentions with a forcible array of evidence from family histories, and with the years his investigations have been extended and the total of his facts amplified. With the ultimate physiological or biochemical determination of sex in the ovum the author does not concern himself. That does not, in his estimation, affect the bare mechanical relativity of male and female. That one ovary supplies a male and the other a female child is the main assertion of his thesis. Anatomical evidence is adduced of the relative size of the ovaries and of the disparity in size and situation of the Fallopian tubes; physiological study bears on the production of ova, on menstruation, and impregnation; pathological investigation on a wide scale in man and other mammalia has been undertaken, to the furnishing of support for the author's reading of events and making of forecasts; and in general a very large field is traversed in varied research, the interpretation ever being to strengthen the main concept of the causation of sex.

The subject is one on which every hygienist should have crystallised ideas. Social conditions make popular the consideration of number and inevitably of sex in families, and the doctor is the natural source of domestic advice in such matters. In this book the subject is treated in a wholesome, intelligent and scientific manner; and whatever may be individual estimates of the completeness of the study and the validity of the inferences, the work deserves the serious attention of every medical man.—A. M.

---

**PULMONARY TUBERCULOSIS.** By David C. Muthu, M.D. 1922. (Baillière, Tindall & Cox; Pp. viii., 381; 12s. 6d. net.)

Among the numerous books published during the last six months on tuberculosis this one is the most original. The author is not afraid to speak plainly, and he denounces the present-day tendency to "echo the catch-phrases, the old shibboleths, and the worn-out opinions of others." He is, perhaps, a little too bold for the stereotyped bacteriologist and the rigid "contagionist" who can only see salvation in notification and segregation of all consumptives. He maintains that tuberculosis is primarily a deficiency disease, a disorder of civilisation, of vicious social and economic environment, and that we have made too much of the tubercle bacillus in the past and too little of man himself.

The recognition of the fact that symptoms of tuberculosis make their appearance long before tubercle bacilli are found in the sputum is strongly urged, and this stage of the disease the author would call physiological, being in accord, as he asserts, with the teachings of biochemistry. He concludes that the bacilli themselves do not create a tuberculous soil, but appear as the result of it. Dr. Muthu shows that by magnifying the dangers of contagion much unnecessary suffering has been inflicted upon consumptives in general.

While there is no royal road to the cure and arrest of tuberculosis, the best results are undoubtedly attained by following Nature's methods. All methods of treatment by injections are, in the opinion of the author, "like working in the dark." He is the great exponent of the mode of treatment by continuous inhalation, and we find a good description of the system as carried out by him for the last twenty years at the Mendip Hills Sanatorium, in which institution, with three good meals a day, the open-air régime is regarded as being the best for the disease.

The broad and truly catholic view of the whole social outlook in relation to tuberculosis taken by the author of this book places it readily in the forefront of those dealing with the "white scourge," and, as such, it may be warmly recommended to all interested in the problem of its eradication.—G. N. M.

---

**TYPICAL FLIES: A PHOTOGRAPHIC ATLAS.** By E. K. Pearce. Second series. 125 illustrations. (Cambridge University Press; 15s. net.)

From the Cambridge University Press comes a well-constructed volume of photographic reproductions of typical flies, an endeavour, according to the author, to complete the series of British types. As in the first series, which appeared in 1915, the photographs are good, and the representation of the flies as truthful as the photographic art can make it. That the atlas can have other than a limited usefulness we cannot say; but the satisfaction we have obtained by comparative study of specimens with the photographs must be felt also by others. In such representation of the flies one hankers after the unreal or embellished—not exactly so, but for the pictorial fixation of the glory of the living creature. The rapidity of desiccation in flies makes photography of the pinned specimen almost futile, except to give a picture of the mummy we possess. A great deal of painstaking and accurate work has gone to the production of this atlas. The notes on habitat, ova, larvæ and distribution are accurate if brief; the measurements almost universally given are valuable to the collector; and while we believe the author may yet see his way to a further publication before the series, in representation of ova and larvæ especially, is completed, we commend the work as a valuable addition to British natural history.—A. M.

---

**A TEXT-BOOK OF GYNÆCOLOGY.** By Jas. Young, D.S.O., M.D., F.R.C.S. (Edin.). (A. and C. Black; 15/- net.)

Textbooks such as this, in which the information is arranged under numerous headings and sub-headings, are beloved of students; for the practitioner such arrangement, while facilitating reference, is apt to bore, since it is like taking meals in tabloid form. Some of the chapters, as, for example, those dealing with menstruation and dysmenorrhœa, are too condensed to be of much practical value to the practitioner, though they may serve to give the student an introduction to the subject. The book is an up-to-date epitome of gynæcology; the illustrations are numerous and of great help in elucidation of the text. The microscopic drawings and those from specimens are excellent.—S. S. F.

